# THE OPIOID EPIDEMIC IN LONG-TERM CARE: A STAFF PERSPECTIVE

**DOI:** 10.1093/geroni/igz038.2604

**Published:** 2019-11-08

**Authors:** Rachael Hemmert, Gabriella E Dull, Linda S Edelman

**Affiliations:** University of Utah, Salt Lake City, Utah, United States

## Abstract

Opioid-based analgesic therapy is a common treatment for moderate to severe pain among long term care (LTC) residents. It has been estimated that 60% of LTC residents have an opioid prescription. Of these, 14% use opioids as part of a long term pain management strategy. LTC residents are particularly vulnerable to opioid misuse, exhibiting higher rates of adverse drug events. However, addressing pain, polypharmacological needs and resident well-being in the LTC setting is challenging. More research and education regarding opioid use in LTC is needed. The Utah Geriatric Education Consortium conducted interprofessional focus groups with LTC partners to 1) determine educational needs of staff regarding opioid use, and 2) gather qualitative data about the pain management experiences of staff when working with residents and families. Staff identified the following training needs: pain manifestation and assessment; certified nurse assistant education on opioid use; non-pharmacological options for pain management. Review of staff’s perception of the intersection of opioids, family and staff in a LTC setting revealed that 1) family is concerned about opioid use; 2) conversely, staff may not see opioid use as a problem; and 3) non-pharmacological options for pain management are often costly and unavailable to those in LTC. Identifying educational needs of LTC staff will help guide the development of educational materials and provide baseline data for future assessments of the impact of opioid education on long-term care patient outcomes.

